# Effectiveness of a five-component multimodal intervention on executive function in children with Autism spectrum disorder: A study protocol for a randomized controlled trial

**DOI:** 10.1371/journal.pone.0345716

**Published:** 2026-03-26

**Authors:** Kazi Md Azman Hossain, Suraiya Yesmin Sharna, Farzana Sharmin, Tofajjal Hossain, Jahid Hasan Naim, Al Amin, Touhidul Islam Badhon

**Affiliations:** 1 Department of Physiotherapy and Rehabilitation, Jashore University of Science and Technology (JUST), Jashore, Bangladesh; 2 Centre for the Rehabilitation of the Paralysed (CRP), Bangladesh; 3 Mymensingh College of Physiotherapy and Health Science, Mymensingh Sadar, Bangladesh; Marwadi University, INDIA

## Abstract

**Background:**

Autism Spectrum Disorder (ASD) is characterized by persistent deficits in social interaction, communication, and executive functions such as inhibitory control, cognitive flexibility, and working memory. These deficits hinder daily functioning and learning outcomes in children. Exercise-based interventions improve executive function; however, most previous studies have focused on single-modality programs with limited generalizability. This trial aims to evaluate the effectiveness of a structured, five-component multimodal intervention—comprising yoga, aerobic, strengthening, neurocognitive, and music-based mindfulness activities—on improving executive functioning in children with ASD.

**Methods:**

This assessor-blinded, double-center randomized controlled trial will enroll 130 children aged 4–18 years diagnosed with ASD in Bangladesh. Participants will be randomly assigned (1:1) to either the experimental group receiving the multimodal intervention or a wait-list control group receiving healthy lifestyle guidelines. The intervention will include five supervised sessions per week for 12 weeks, followed by a 12-week follow-up. An additional group of 65 typically developing children will serve as healthy controls. Primary outcomes will assess executive function domains: inhibitory control (Go/No-Go Task), cognitive flexibility (Trail Making Test A–B), and working memory (Corsi Block Tapping Task; Forward and Backward Digit Span). Secondary outcomes include social responsiveness (Social Responsiveness Scale–2) and ASD-related behaviors (Autism Treatment Evaluation Checklist). Outcomes will be evaluated at baseline, 12 weeks, and 24 weeks by blinded assessors. Data will be analyzed using SPSS following the intention-to-treat principle.

**Discussion:**

This trial will address an important evidence gap by evaluating a comprehensive, low-cost, non-pharmacological multimodal intervention targeting multiple domains of executive function in children with ASD. The findings may help clinicians, educators, and policymakers adopt structured exercise-based programs within rehabilitation, school, and community settings. Although limited to two centers, the results could guide future large-scale studies and support the development of standardized multimodal intervention guidelines for ASD.

**Trial registration:**

This trial is prospectively registered with the Clinical Trial Registry of India: CTRI/2025/11/096943 [Registered on: 06/11/2025]. Link: https://ctri.nic.in/Clinicaltrials/pmaindet2.php?EncHid=MTQ1NDEy&Enc=&userName=

## Introduction

Autism Spectrum Disorder (ASD) is a neurodevelopmental condition that usually presents from early childhood, marked by social impairments and repetitive, limited behaviors, hobbies, or activities. Diagnosis often occurs between the ages of 3 and 10, with a mean age of diagnosis of 4 to 5 years. The incidence of ASD is widely acknowledged as a critical public health concern, impacting children’s health worldwide [1,2]. The World Health Organization reported that around 1 in 100 children and about 1% of the global population are affected by ASD [3,4]. The prevalence of ASD in Bangladesh varies from 0.15% to 0.8%, with accuracy influenced by factors including age, diagnostic criteria, geographical region, perinatal age, maternal age, and the availability of awareness and resources for persons with ASD [5]. In this regard, monitoring the health and well-being of persons with ASD is essential, although it is a difficult task. The extensive diversity of symptoms and comorbidities linked to this illness hinders management, as demonstrated by the presence of many health issues concurrently. Sleep difficulties, metabolic abnormalities, motor impairments, gastrointestinal issues, and obesity are recognized as the predominant comorbidities among the ASD community. Individuals with ASD have markedly reduced levels of physical activity compared to their peers without the disorder [6].

Executive functions are higher-order cognitive skills that enable planning, decision-making, emotional regulation, and self-control. Children with ASD often face challenges in these areas, including executive functions. The three primary elements of executive functioning are inhibitory control, cognitive flexibility, and working memory. Inhibitory control regulates impulses; when deficient, children may exhibit impulsive behavior or encounter social difficulties. Cognitive flexibility facilitates the transition between activities or concepts; deficiencies in this domain might hinder adaptability to change. Working memory facilitates the retention and manipulation of information; its limitations may impede the execution of complex activities or the retrieval of details. These issues affect every aspect of life for children with ASD; nevertheless, augmenting executive functioning can enhance their flexibility and overall growth. Growing evidence suggests that exercise-based interventions can enhance cognitive, emotional, and social abilities, as well as overall executive functions, in children with ASD [7,8]. It provides a cost-effective, accessible, and less detrimental alternative to conventional therapy. Individual or small-group exercise programs can enhance executive functioning and quality of life in children with ASD. It can also replace non-targeted stereotyped behaviors, reducing reliance on these behaviors for fulfilment [9].

An increasing amount of evidence indicates that several exercise-based interventions can markedly enhance executive function deficiencies in children with ASD. Structured programs, including yoga, aerobic exercise, strengthening exercise, neurocognitive tasks, and music-based interventions, have shown measurable benefits for attention, emotional regulation, social interaction, and cognitive flexibility [7,8]. Yoga-based interventions enhance attention, self-regulation, and social communication by integrating breathing, movement, and mindfulness within fun, structured environments [10]. Aerobic exercise improves executive functioning via neurobiological processes, such as elevated brain-derived neurotrophic factor (BDNF) levels and stimulation of the BDNF/TrkB signaling pathway, hence fostering neuroplasticity and cognitive advancements [7]. Strengthening exercises are essential, as increased physical strength correlates with improved working memory, inhibitory control, and overall cognitive efficiency [11]. Likewise, neurocognitive sports activities stimulate substantial brain areas, especially the prefrontal cortex, resulting in enhanced attention, flexibility, and adaptive behavior [12]. Furthermore, musical intervention enhances attention, working memory, and emotional regulation by activating brain networks associated with social cognition and executive control [13]. These approaches together demonstrate that focused physical and mindfulness-based activities can significantly enhance the development of executive functions and adaptive behaviors in children with ASD [7–13].

Current research on exercise interventions for children with ASD is constrained by an emphasis on single-modality programs, lacking a systematic integration of diverse exercise modalities to target executive function deficiencies. Systematic reviews and meta-analyses suggest that although physical activity has a positive impact on autism severity, social communication, motor performance, and cognition, there are deficiencies in addressing the complex aspects of executive function, optimal dosage, intervention intensity, and individual variability [7]. In this regard, this study will implement a 12-week, five-component multimodal intervention comprising yoga, aerobic exercise, strengthening exercise, neurocognitive tasks, and a music-based intervention, aimed at simultaneously addressing cognitive, emotional, and physical dimensions. This holistic approach aims to enhance executive functioning through comprehensive intervention programs, small-group sessions, personalized development, and caregiver participation to optimize motivation, social skills, and family support in children with ASD.

## Methods

### Study design

**Fig 1:**
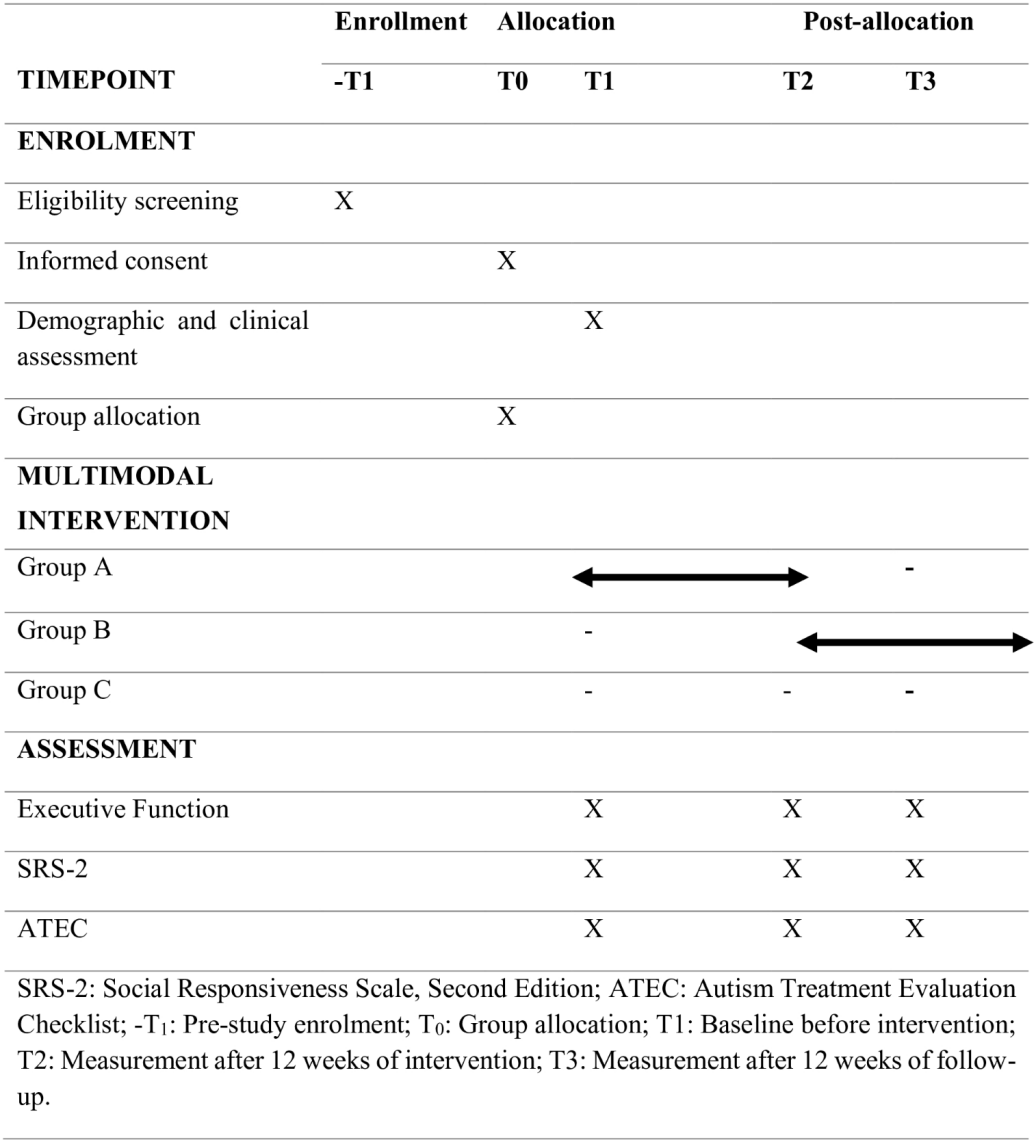
SPIRIT guideline.

**Fig 2:**
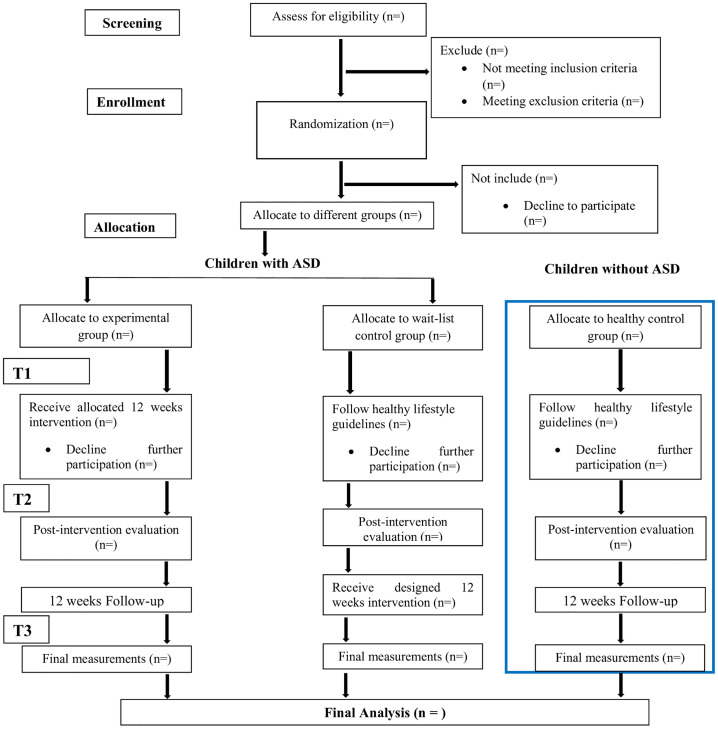
CONSORT flow diagram.

We will conduct a double-center, assessor-blinded, randomized controlled trial to assess the efficacy and safety of a five-component multimodal intervention for children diagnosed with ASD. The study will involve 130 children with ASD, randomly assigned to two groups: a multimodal intervention (experimental: group A) and a healthy lifestyle guidelines (wait-list control: group B). The intervention will last 12 weeks. We will also recruit 65 healthy children as healthy controls (Group C), who will not receive multimodal intervention and will follow healthy lifestyle guidelines. All participants will be assessed at baseline (T1), immediately after the intervention (T2), and after a 12-week follow-up (T3). The assessments will focus on executive functions, social responsiveness, and ASD-related characteristics, with a primary emphasis on safety and tolerability. This study protocol complies with the SPIRIT (Fig 1) and CONSORT (Fig 2) guidelines.

### Ethical considerations

A participant information sheet will be provided, and informed consent will be obtained from the parents or caregivers of participants. The study protocol has been reviewed and approved by the Institutional Review Board of the Department of Physiotherapy and Rehabilitation at Jashore University of Science and Technology (ID: PTR-JUST/IRB/2025/10/192404). This trial is registered as a randomized controlled trial in the Clinical Trials Registry-India (CTRI); registration number: CTRI/2025/11/096943, registered on 6 December 2025. The study will follow the Declaration of Helsinki, Good Clinical Practice guidelines, and all relevant regulatory standards. To ensure proper oversight, key study personnel will periodically review trial progress, including participant enrollment, protocol compliance, data accuracy, and adverse events. Because of the low-risk nature of the multimodal intervention, a formal independent data monitoring committee will not be established. Instead, the study team will oversee safety and data integrity internally. All significant adverse events or protocol deviations will be reported to the Ethics Committee in accordance with regulatory standards. No interim analyses or independent audits are planned. Any major protocol updates will be reviewed by the Principal Investigator and submitted to the Ethics Committee for approval. Once approved, all team members will be notified, and the updated protocol will be documented in the study records. Deviations from the approved protocol will be documented with a breach report form, and the Clinical Trial Registry will be amended as necessary.

### Study settings

The study will be conducted at the Paediatrics laboratory within the Department of Physiotherapy and Rehabilitation at Jashore University of Science and Technology, and Proyash, Jashore, Bangladesh. Additional collaboration will involve community health clinics and associations supporting children with ASD to recruit targeted participants. Interventions will be delivered in person, as outlined in the study protocol, on an outpatient basis at the two study sites.

### Sample size calculation

The required sample size was determined using G*Power (v.3.1.9.7; University of Kiel, Germany) for a 2 × 3 mixed-model ANOVA (Group × Time interaction) across three assessment points (T1, T2, T3). Assuming a two-tailed test, an anticipated effect size of 0.475 based on prior executive functioning research [8], an α significance level of 0.05, and a power (1 − β) of 0.80, the analysis indicated that a minimum of 112 participants with ASD were required. To accommodate an expected attrition rate of 15%, the recruitment target was increased to 130 participants with ASD, 65 per group. In addition, 65 age- and gender-matched healthy participants will be recruited to serve as a reference group for baseline and exploratory comparisons and were not included in the power calculation. Although the primary outcomes are multivariate, sample size estimation was based on the most conservative univariate effect to ensure adequate statistical power; correlations among outcomes and baseline differences will be addressed using multivariate analytical approaches.

### Participants recruitment and data collection

A total of 130 children with ASD will be recruited through a combination of online and offline advertisements, public announcements at religious institutions, and flyers distributed in the surrounding areas of Jashore, Bangladesh. Additionally, 65 healthy children will be recruited from two schools in Jashore, aligning with the demographic characteristics of children with ASD. Interested individuals who meet the eligibility criteria will be invited to attend an informational session that explains the study’s objectives, procedures, potential risks, and benefits, followed by an interactive question-and-answer discussion at the study sites. Written informed consent will be obtained from parents or caregivers prior to enrolment. Participation will be entirely voluntary, and individuals may withdraw from the study at any time without affecting their treatment. We will use REDCap (Research Electronic Data Capture) to manage clinical data. Personal information will be kept separate from study data, stored securely on password-protected systems, and pseudo-anonymized using unique numeric codes that are accessible only to authorized personnel. The recruitment and data collection processes will ensure fairness, transparency, and impartiality in participant selection.

### Eligibility criteria

Qualified health professionals will recruit participants through an impartial initial screening process guided by the study’s inclusion and exclusion criteria.

#### Inclusion criteria.

Participants will be included if they are aged between 4 and 18 years, including both boys and girls, who have been diagnosed with ASD in accordance with the American Diagnostic and Statistical Manual of Mental Disorders (DSM-5) [7], based on clinical assessment and confirmed by evaluation using the Childhood Autism Rating Scale, Second Edition (CARS-2), conducted by a child psychiatrist and consent to participate in a 12-week intervention period [14].

#### Exclusion criteria.

Participants will be excluded if they have cerebral palsy, Down syndrome, or attention deficit/hyperactivity disorder (ADHD). Additionally, any participant who reports, or whose parent reports, a recent (within the past six months) muscle injury or other conditions—such as muscle sprain, head injury, or skin laceration—that may interfere with study outcomes or exercise participation [15].

Additionally, the children in the healthy control group will undergo the same interview as children with ASD to confirm they are typically developing and will complete the same outcome measures as the ASD group, with the aim of investigating whether the ASD group can reach normal developmental levels on outcome measures after the intervention.

### Randomization and blinding

Participants will be randomly assigned to either the experimental group or the wait-list control group in a 1:1 ratio using a computer-generated sequence with varying block sizes, stratified by site (two sites). Randomization will be centrally managed by the Clinical Trials Unit (CTU) through a secure web-based system, with allocation concealed from investigators. Due to the nature of the intervention, blinding of parents and therapists will not be feasible; however, outcome assessors will remain blinded to group allocation. Research staff, statisticians, and investigators involved in data analysis will also remain blinded.

### Outcome measurements

All sociodemographic and related clinical characteristics will be measured at baseline (T1). The primary and secondary outcome measures will be assessed at three time points: at baseline (T1), immediately after the intervention (T2), and following a 12-week follow-up period (T3) by six blinded assessors. All primary and secondary outcomes are listed below with brief descriptions.

#### Primary outcomes.

The primary outcome will be Executive Function, assessed through three subcomponents: Inhibitory Control, Cognitive Flexibility and Working Memory. Inhibitory Control (IC) will be measured using the Go/No-Go (GNG) task; Cognitive Flexibility will be assessed with the Trail Making Test (TMT); Working Memory (WM) will be evaluated using the Corsi Block Tapping Task (CBTT), Forward Digit Span (FDS) test, and Backward Digit Span (BDS) test [16,17].

#### Inhibitory control: Go/No-Go (GNG) task.

Inhibitory control will be evaluated using a computer-based Go/No-Go (GNG) task. Participants will sit in front of a computer monitor and press the left or right arrow key when the corresponding arrow appears (Go trials) and withhold responses when an upward arrow appears (No-go trials). After 20 practice trials, they will complete 300 experimental trials (220 Go and 80 No-go), each presented for 500 ms, with a 1000 ms interstimulus interval, using E-Prime 3.0 software. A false alarm—pressing a key during a No-go trial—will indicate reduced inhibitory control. Participants who respond correctly to fewer than 50% of Go trials will be excluded from the analysis.

#### Cognitive flexibility: Trail Making Test (TMT).

Cognitive flexibility will be assessed using a computer-based Trail Making Test (TMT). The task will consist of two parts: Part A (numbers only) and Part B (numbers and letters), each with a single trial. Before each trial, participants will be instructed to complete Part A by connecting numbers in sequence (e.g., 1, 2, 3… up to 25) and Part B by alternating between numbers and corresponding letters in numerical and alphabetical order (e.g., 1-A, 2-B, 3-C… up to 13) using a mouse. Performance will be measured by the number of errors and the total time taken to complete each part. Shorter completion times and fewer errors will indicate better cognitive flexibility.

#### Visual-spatial working memory: Corsi Block Tapping Task (CBTT).

Visual-spatial working memory will be assessed using the Corsi Block Tapping Task. Participants will observe a sequence of blocks being tapped and will be instructed to reproduce the sequence in the same order. The sequence will start with three blocks and will increase by one after every four trials. The task will end after four consecutive incorrect repetitions at the same sequence length, and the longest correctly reproduced sequence will be recorded as the participant’s score.

#### Auditory working memory: Forward and backward digit span tests.

Auditory working memory will be assessed using the Forward Digit Span (FDS) and Backward Digit Span (BDS) tests. Digits will be presented at a rate of one per second, and participants will be asked to verbally repeat each sequence either in the same order (FDS) or in reverse order (BDS). The sequence will begin with two digits and increase by one after every two trials. Testing will end after two consecutive errors at the same sequence length, and the maximum digit span will be recorded.

### Secondary outcomes

The secondary outcomes will be social responsiveness and autism-related behavioral changes. Social responsiveness will be assessed using the Social Responsiveness Scale, Second Edition (SRS-2) [18], and ASD-related characteristics will be evaluated using the Autism Treatment Evaluation Checklist (ATEC) [19].

#### Social responsiveness scale, Second Edition (SRS-2).

The Social Responsiveness Scale, Second Edition (SRS-2) will be administered to assess social responsiveness in participants. This is a valid measure and has demonstrated reliability and feasibility, providing meaningful insights for children with ASD. The scale comprises 65 items across five subscales: Social Awareness (8 items), Social Cognition (12 items), Social Communication (22 items), Social Motivation (11 items), and Behavior Pattern (12 items). In this study, the caregiver of participants will complete the SRS-2, with the same person conducting the assessment at T1, T2, and T3. Scoring will include the total score and the five subscale scores, with higher scores indicating greater severity of social responsiveness impairment.

#### Autism Treatment Evaluation Checklist (ATEC).

The Autism Treatment Evaluation Checklist (ATEC) will be administered as a caregiver-reported questionnaire to assess changes in ASD-related characteristics following the intervention. The ATEC provides a total score and four subscale scores. The first three subscales—Speech/Language/Communication, Sociability, and Sensory/Cognitive Awareness—will be scored on a 0–2 scale, while the fourth subscale, Health/Physical/Behavior, will be scored on a 0–3 scale. Scores from all subscales will be combined to calculate a total score ranging from 0 to 179, with lower scores indicating reduced severity of ASD-related characteristics and higher scores indicating greater severity.

### Intervention details

On the first day, an assessor blinded to group assignment will conduct additional screening and administer the pre-intervention baseline evaluation. Participants will then receive an intervention based on their group allocation. All participants will receive a booklet outlining healthy lifestyle guidelines and be advised to follow them throughout the entire 24-week study period. Afterwards, wait-list control group participants will receive additional guidelines for the 12-week intervention phase. During this period, a biweekly discussion session will be organized for control group participants. After the 12-week intervention, the wait-list control group will also receive the intervention provided to the experimental group.

Participants in the experimental group will undergo a 12-week multimodal intervention led by experienced and trained physiotherapists. The intervention methods, dosages and progression plans will be meticulously monitored by four research assistants to ensure consistency across all participants in the intervention groups.

#### Five-component multimodal intervention (Experimental group).

The intervention will consist of five sessions per week, held in small groups of five participants, each lasting 60 minutes. It incorporates five evidence-based components designed to address the multidimensional needs of children with ASD—physical fitness, cognitive engagement, emotional regulation, and social participation. Each session will include 10 minutes of yoga to enhance flexibility and self-regulation [20]; 15 minutes of aerobic activity to improve cardiovascular fitness and mood stability [17]; 10 minutes of strengthening exercises targeting major muscle groups [21]; 15 minutes of neurocognitive tasks promoting attention and executive functioning [17]; and 10 minutes of music-based mindfulness to support relaxation and social interaction [20]. The program will progressively adapt to individual ability levels to ensure safety, engagement, and sustainable improvement in overall outcomes [22]. Detailed information on interventions is reported in Table 1.

**Table 1:** Five-component multimodal intervention.

Exercise component	Exercise description
Yoga 10	− 5 min guided meditation and deep breathing − 5 min static and dynamic poses (Mountain, Rag doll, Tree, Cat–Cow, Child’s, Happy baby, Corpse Pose)
Aerobic exercise 15 min	- Skipping rope - Agility ladder drills - Cardio kickboxing - Low-impact aerobics relay games 10 reps/1–3 sets; progress to complexity (directional changes, faster tempo)
Strengthening exercise 10 min	- Body-weight squats - Wall push-ups - Step-ups (15 cm platform) - Resistance-band exercise focusing on major muscle groups 10 reps/1–3 sets
Neurocognitive tasks 15 min	- Color-response and dual-task games (e.g., passing the ball while counting) - Table tennis/badminton with rule-based challenges 4–5 tasks/1–3 sets; progress to reducing reaction time, adding extra cues, or increasing multitasking complexity
Music-based mindfulness 10 min	- Listening to familiar songs (“Little Star,” “Red Dragonfly,” “Jingle Bells,” “Wahaha”) - Guided rhythm imitation (clapping, tapping, swaying) 2–3 rhythm cycles; progress to participant-led rhythm creation

Each exercise will include a 15-second rest period between sets and a 1-minute rest interval between different components of the intervention. Participants will attend five supervised sessions per week for 12 weeks, each lasting approximately 60 minutes.

### Follow‑up

A follow-up evaluation will be conducted 12 weeks after the last intervention session for the experimental groups to assess the persistence of the intervention’s effectiveness. During these 12 weeks, all participants will be instructed to follow the previous healthy lifestyle guidelines, and the control group participants will receive the experimental group interventions. There will be two discussion sessions in the meantime for all participants, following the condition and health status of children with ASD.

### Intervention fidelity (therapist manual fidelity)

Intervention fidelity will be maintained through mandatory monthly supervision of all participating therapists, ensuring adherence to the intervention manual. Any deviations from the prescribed protocol will be quickly identified and rectified. All intervention sessions will be recorded in a centralized database for later analysis of treatment adherence, and fidelity outcomes will be included in the study manuscripts. Additionally, four trained research assistants will observe and document each session to verify the accuracy of implementation and protocol compliance.

### Safety measures and adverse event management

Before enrolment, participants will undergo screening to confirm eligibility and exclude risk factors. A qualified staff member will review medical history and conduct a physical examination. During the multimodal exercise program, sessions will be supervised by a trained physiotherapist and monitored by the principal investigator and study team. Participants’ health status and any discomfort will be checked before each session. Any adverse events, though expected to be minimal, will be documented in SOAP notes and reported to the principal investigator. Participants will receive appropriate treatment based on the nature and severity of any adverse effects. The principal investigator and monitoring staff will collaboratively manage safety concerns and report significant events to the Institutional Review Board. Continuous monitoring will ensure participant safety throughout the program.

### Statistical analysis

Statistical analysis will be conducted using SPSS version 26. Continuous variables will be summarized as mean ± standard deviation (SD), and categorical variables as frequencies and percentages. Baseline differences across the three groups (two ASD intervention groups and healthy controls) will be examined using one-way ANOVA for normally distributed variables and Kruskal–Wallis tests for non-normally distributed data. Primary intervention effects across three assessment points (T1, T2, T3) will be evaluated using linear mixed-effects models (LMMs), with group specified as the between-subjects factor and time as the within-subjects factor, and pre-specified baseline covariates (age, sex, and body mass index). All covariates were measured at baseline prior to randomization and were included based on their known associations with executive function; post-randomization or time-varying covariates were not adjusted for to avoid mediation or over-adjustment bias. Although sample size estimation was based on mixed-model ANOVA, LMMs were selected for primary analysis due to their robustness to missing data and flexibility in modelling repeated measures. Model assumptions, including normality and homoscedasticity, will be assessed, and robust or rank-based alternatives will be applied if violations are detected. Significant group × time interactions will be explored using simple main effects with Holm–Bonferroni correction for multiple comparisons. Effect sizes will be reported as partial η² for mixed models and Cohen’s d (pooled SD) for pairwise comparisons, interpreted using conventional benchmarks. Within-group changes across time points will be examined using paired-samples t-tests, interpreted in relation to both statistical significance (p < 0.05) and established minimal clinically important differences (MCID) where available. To control family-wise error across multiple primary outcomes, Holm–Bonferroni adjustment will be applied. Missing data will be handled under the assumption of missing at random (MAR) using an intention-to-treat approach with multiple imputation (20 imputed datasets generated via fully conditional specification, including all outcomes and covariates). Sensitivity analyses will be performed to explore the potential impact of outcome-dependent exclusions, such as participants with fewer than 50% correct responses on Go trials. Multivariate relationships among primary outcomes will be further examined using multivariate analysis of covariance (MANCOVA), adjusting for baseline covariates and assessing assumptions of multivariate normality and homogeneity of covariance matrices. This analytical strategy ensures robust, unbiased, and clinically interpretable inference in longitudinal, multigroup studies.

### Confidentiality

All personal information of participating children and their caregivers will remain strictly confidential. Any photographs or videos captured during training sessions will have the child’s face blurred to ensure anonymity. Evaluation forms and other study records will be labelled only with a unique coded number, allowing data to be tracked without revealing participant identities. All records will be stored securely, and data access will be limited to these coded identifiers. No clinical information will be shared without the prior written consent of the child’s legal guardian.

### Dissemination policy

The study findings, based on de-identified individual data, will be disseminated through publications in international peer-reviewed journals, conference presentations, and relevant Bangladeshi media and institutional websites. Study funders will be appropriately acknowledged in all publications and public communications. Manuscripts will be prepared by the research team, comprising academics, researchers, and research assistants, in accordance with the Vancouver authorship guidelines. Both positive and negative results will be published to ensure transparency. Key experiences related to the intervention’s implementation and evaluation will also be shared with relevant academic, clinical, and community stakeholders.

## Discussion

ASD is a complex neurodevelopmental condition that significantly affects social functioning, adaptive behaviors, and executive functions in children. Executive functions are essential for planning, problem-solving, and adaptive behavior. Impairments in these domains can limit educational achievement, daily living activities, and social participation in children with ASD [23]. Evidence suggests that interventions targeting these cognitive domains may promote developmental progress and improve quality of life. Exercise-based interventions have emerged as a promising nonpharmacological approach to enhance executive functioning, social skills, and emotional regulation in children with ASD [7,23].

This study investigates a 12-week, five-component multimodal exercise-based intervention, integrating yoga, aerobic, strengthening, neurocognitive, and music-based activities, to improve executive function in children with ASD. By combining multiple modalities, this approach addresses cognitive, emotional, social, and physical domains simultaneously, potentially offering advantages over single-modality interventions. Previous studies have shown the effectiveness of these five types of intervention when applied as a single treatment [17,20,21]. The multimodal design of this intervention offers structured yet flexible programming, enabling adaptations to individual abilities and ensuring engagement, safety, and sustained improvements. Small-group settings facilitate peer interaction and social learning while maintaining personalized attention from trained physiotherapists. Additionally, caregiver involvement may enhance motivation, adherence, and the generalization of skills to home and school environments, addressing gaps in previous research that often neglect parental participation. The inclusion of a wait-list control group and matched healthy controls enables a rigorous evaluation of intervention efficacy and developmental gains in comparison to typical trajectories [17].

This study may have important implications for clinical practice and public health for children with ASD in Bangladesh and worldwide. Positive outcomes could inform the development of evidence-based, scalable, and accessible multimodal exercise protocols for children with ASD, addressing both cognitive and social deficits. Such interventions are cost-effective, non-invasive, and easily integrated into school or community programs, offering an alternative to conventional therapies that may be less accessible due to financial, geographic, or resource constraints. Furthermore, demonstrating sustained effects at the 12-week follow-up would support the long-term adoption of these practices and encourage self-management strategies for children and their families.

Nevertheless, several limitations need to be acknowledged. While assessor blinding reduces bias, parents and therapists cannot be blinded due to the nature of the intervention, which may lead to expectancy effects. The study population is limited to specific sites in Jashore, Bangladesh, which might affect its applicability to broader regional or national populations. Attrition during the intervention or follow-up period may impact statistical power and the interpretation of long-term effects. Future research could consider larger multicenter trials, different dosage regimens, and long-term neurodevelopmental outcomes, including academic performance and adaptive behavior, to enhance the evidence base.

In conclusion, this study addresses a significant gap in ASD research by examining the effects of a five-component multimodal exercise-based intervention on executive function and related outcomes in children with ASD. By providing a comprehensive, evidence-based, and feasible intervention, this study has the potential to improve cognitive, social, and adaptive skills, enhancing overall quality of life. Findings may inform practitioners, educators, and policymakers, supporting the implementation of community- and school-based programs that promote developmental gains in children with ASD.

### Trial status

This study will begin recruiting participants in December 2025 and is expected to finish with follow-up evaluation and be prepared for publication by August 2026.

### Patient and public involvement

Patients and/or the public were not involved in the design, conduct, reporting, or dissemination plans of this research.

## Supporting information

S1 File SPIRIT checklist. (DOCX)

S2 File EC approved study protocol. (PDF)
