# Supplementary material for: Effectiveness of a five-component multimodal intervention on executive function in children with Autism spectrum disorder: A study protocol for a randomized controlled trial
Source: PLoS One. 2026 Mar 26;21(3):e0345716. doi: 10.1371/journal.pone.0345716 (PMC13020812; doi:10.1371/journal.pone.0345716)
Supplement: S2 File — (PDF) [file pone.0345716.s002.pdf]

**Institutional Review Board (IRB)**
**Department of Physiotherapy & Rehabilitation**
**Jashore University of Science & Technology**
**mail:** chairman.ptr@just.edu.bd **website:** https://ptr.just.edu.bd/

# PROPOSED STUDY PROTOCOL

Date: 08/10/2025
Institutional Review Board (IRB)
Department of Physiotherapy & Rehabilitation
Jashore University of Science & Technology
Jashore-7408, Bangladesh

Subject: Submission of documents for study title, **"Effectiveness of a Five-Component Multimodal Intervention on Executive Function in Children with Autism Spectrum Disorder: A Randomized Controlled Trial".**

Dear Sir,

I hereby submit the documents for the referenced study for your review and approval to proceed. The following list of documents was submitted for your review -

| SL No | Documents | Version | No of copies |
|---|---|---|---|
| 1 | Ethical Statement Checklist | 1 | 1 |
| 2 | Research Methodology | 1 | 1 |
| 3 | Participant Information Sheet (PIS) | 1 | 1 |
| 4 | Informed Consent Form | 1 | 1 |
| 5 | Questionnaire | 1 | 1 |
| 6 | Intervention Tracking Sheet/ Checklist | 1 | 1 |
| 7 | Adverse Effect Tracking and Management Form | 1 | 1 |
| 8 | Time Frame: (Gantt Chart) | 1 | 1 |
| 9 | Budget | 1 | 1 |
| 10 | Funding Document | 1 | 1 |

EC Approved Protocol

Requesting acknowledgement of the submission letter and its enclosures. Please let us know if any further information or clarification is required.
Looking forward to your approval.

Yours sincerely,
Kazi Md Azman Hossain
Physiotherapist
Department of Physiotherapy & Rehabilitation
Jashore University of Science & Technology
Jashore-7408, Bangladesh

EC Approved Protocol

**Approved on**
**October 22, 2025**

**Institutional Review Board (IRB)**
**Department of Physiotherapy & Rehabilitation**
**Jashore University of Science & Technology**
**mail:** chairman.ptr@just.edu.bd **website:** https://ptr.just.edu.bd/

## 1. Annexure: A – Ethical Statement Checklist

### Put a tick sign (√) appropriate answers against each of the following statements (If not Applicable, please write N/A)

**1. Source of Population:**

a) Patients ☑ Yes ☐ No
b) Healthy Subjects ☑ Yes ☐ No
c) Minors or persons under guardianship ☑ Yes ☐ No

**2. Does the study involve:**

a) Physical risks to the Subjects ☐Yes ☑ No
b) Social Risks ☐Yes ☑ No
c) Psychological risks ☐Yes ☑ No
d) Discomfort to the subject ☐Yes ☑ No
e) Invasion of the body ☐Yes ☑ No
f) Invasion of Privacy ☐Yes ☑ No
g) Disclosure of information damaging to the subject or others ☐Yes ☑ No

**3. Does the study involve:**

a) Use of records: - (Hospital, Medical, Death, Birth, or other) ☑Yes ☐ No
b) Use of fetal tissues
c) or abortus ☑Yes ☐ No
Use of organs or body fluids ☐ Yes ☑ No

**4. Will subjects inform about:**

a) Nature and purposes of study ☑ Yes ☐ No
b) Procedures to be followed including alternative used ☑ Yes ☐ No
c) Physical risks ☑ Yes ☐ No
d) Private questions ☑ Yes ☐ No
e) Mental risks ☑ Yes ☐ No
f) Benefits to be derived ☑ Yes ☐ No
g) Right to refuse to participate or to withdraw from study ☑ Yes ☐ No
h) Confidential handling of data ☑ Yes ☐No
i) Compensations: (where there are risks or loss of working time or privacy is involved in any particular procedure) ☐ Yes ☑ No

**5. A signed consent form will be obtained:**

a) From Subjects (If adult) ☑ Yes ☐ No
b) From parent or guardian ☑ Yes ☐ No

**6. Will precautions be taken to protect the anonymity of subjects?**
☑ Yes ☐ No

EC Approved Protocol

## 2. Annexure: B – Research Methodology

| | |
|---|---|
| **Research Title** | Effectiveness of a Five-Component Multimodal Intervention on Executive Function in Children with Autism Spectrum Disorder: A Randomized Controlled Trial |
| **Eligibility criteria** | Qualified health professionals will recruit participants through an impartial screening process that adheres to predefined inclusion and exclusion criteria. Children aged 4–18 years of both sexes with a confirmed ASD diagnosis according to DSM-5 and CARS-2, assessed by a child psychiatrist, and who consent to participate in a 12-week intervention will be included. Participants will be excluded if they have cerebral palsy, Down syndrome, ADHD, or any recent (past six months) muscle injury, sprain, head injury, skin laceration, or other conditions that may hinder exercise or study outcomes. Children in the healthy control group will undergo the same interview to confirm typical development and will complete the same outcome measures to compare whether the ASD group can achieve normal developmental levels after the intervention. |
| **Study setting** | Paediatrics laboratory within the Department of Physiotherapy and Rehabilitation at Jashore University of Science and Technology, and Proyash, Jashore, Bangladesh. |
| **Date of recruitment** | 10/12/2025 |
| **Study method** | Randomized Controlled Trial |
| **Reporting guideline** | CONSORT |
| **Intervention details** | All participants will receive a healthy-lifestyle guideline booklet to follow throughout the 24-week study. The wait-list control group will receive these guidelines plus biweekly discussion sessions during their initial 12-week period and will later receive the same intervention as the experimental group. The experimental group will complete a 12-week, five-component multimodal program delivered by trained physiotherapists, with |

EC Approved Protocol

| | |
|---|---|
| | all procedures, dosages, and progressions monitored by four research assistants for consistency. Each 60-minute session, conducted five times per week in small groups, will include yoga (10 minutes), aerobic exercise (15 minutes), strengthening exercises (10 minutes), neurocognitive tasks (15 minutes), and music-based mindfulness (10 minutes), with structured rest intervals and progressive adjustments based on individual abilities. Detailed intervention content is presented in a supplementary file. A follow-up assessment will occur 12 weeks after the intervention to evaluate sustained effects, during which all participants will continue following lifestyle guidelines, and the control group will undergo the experimental intervention, accompanied by two discussion sessions addressing the health needs of children with ASD. |
| **Intervention provider** | Experienced and qualified health professionals |
| **Outcome measurement** | *Primary outcomes*<br>The primary outcome will be Executive Function, assessed through three subcomponents: Inhibitory Control, Cognitive Flexibility and Working Memory. Inhibitory Control (IC) will be measured using the Go/No-Go (GNG) task; Cognitive Flexibility will be assessed with the Trail Making Test (TMT); Working Memory (WM) will be evaluated using the Corsi Block Tapping Task (CBTT), Forward Digit Span (FDS) test, and Backward Digit Span (BDS) test.<br>*Secondary outcomes*<br>The secondary outcomes will be social responsiveness and autism-related behavioral changes. Social responsiveness will be assessed using the Social Responsiveness Scale, Second Edition (SRS-2), and ASD-related characteristics will be evaluated using the Autism Treatment Evaluation Checklist (ATEC) |
| **Statistical analysis** | Statistical analysis will be conducted using SPSS version 26. Continuous variables will be summarized as mean ± standard deviation (SD), and categorical variables as frequencies and percentages. Baseline differences across the three groups (two ASD intervention groups and healthy controls) will be examined using one-way ANOVA for normally distributed variables and |

EC Approved Protocol

| | |
|---|---|
| | Kruskal–Wallis tests for non-normally distributed data. Primary intervention effects across three assessment points (T1, T2, T3) will be evaluated using linear mixed-effects models (LMMs), with group specified as the between-subjects factor and time as the within-subjects factor, and pre-specified baseline covariates (age, sex, and body mass index). All covariates were measured at baseline prior to randomization and were included based on their known associations with executive function; post-randomization or time-varying covariates were not adjusted for to avoid mediation or over-adjustment bias. Although sample size estimation was based on mixed-model ANOVA, LMMs were selected for primary analysis due to their robustness to missing data and flexibility in modelling repeated measures. Model assumptions, including normality and homoscedasticity, will be assessed, and robust or rank-based alternatives will be applied if violations are detected. Significant group × time interactions will be explored using simple main effects with Holm–Bonferroni correction for multiple comparisons. Effect sizes will be reported as partial $\eta^2$ for mixed models and Cohen's d (pooled SD) for pairwise comparisons, interpreted using conventional benchmarks. Within-group changes across time points will be examined using paired-samples t-tests, interpreted in relation to both statistical significance ($p < 0.05$) and established minimal clinically important differences (MCID) where available. To control family-wise error across multiple primary outcomes, Holm–Bonferroni adjustment will be applied. Missing data will be handled under the assumption of missing at random (MAR) using an intention-to-treat approach with multiple imputation. Sensitivity analyses will be performed to explore the potential impact of outcome-dependent exclusions, such as participants with fewer than 50% correct responses on Go trials. Multivariate relationships among primary outcomes will be further examined using multivariate analysis of covariance (MANCOVA), adjusting for baseline covariates and assessing assumptions of multivariate normality and homogeneity of covariance matrices. This analytical strategy ensures robust, unbiased, and clinically interpretable inference in longitudinal, multigroup studies. |

EC Approved Protocol

| | |
|---|---|
| **Principal investigator (s)** | Kazi Md Azman Hossain<br>Department of Physiotherapy and Rehabilitation, JUST<br>Email: azmanhossain51@gmail.com |
| **Funding source (s)** | This study received partial funding for data collection and intervention from the University Grants Commission through the Physiotherapy and Rehabilitation Department, Jashore University of Science and Technology (JUST), Jashore, Bangladesh. |
| **Ethical considerations** | All participants will provide written informed consent before enrollment. The study will adhere to the ethical guidelines outlined in the Declaration of Helsinki. |

EC Approved Protocol

# 3. Annexure: C – Participant Information Sheet (PIS)

**Department of Physiotherapy and Rehabilitation**
**Jashore University of Science and Technology**

## Growing Healthy Together: A Wellness Initiative for Children

**Free Physiotherapy Intervention for Children with and without ASD**
**Participants will receive free physiotherapy treatment for 12 weeks**

| Inclusion Criteria | Exclusion Criteria |
|---|---|
| Children aged 4–18 years of both sexes | Diagnosis of Cerebral Palsy or Down Syndrome |
| Confirmed ASD diagnosis (DSM-5 and CARS-2) Or, Possible ASD based on their symptoms | Diagnosis of ADHD |
| Consent to participate in a 12-week intervention | Recent (past 6 months) muscle injury, sprain, or head injury |
| Typical development (for the healthy control group) | Skin lacerations or conditions hindering exercise |

| Intervention Sites | |
|---|---|
| Paediatrics laboratory<br>Department of Physiotherapy and Rehabilitation<br>Jashore University of Science and Technology<br>Jashore, Bangladesh | Proyash<br>Jashore, Bangladesh |

| What Will Participants Receive? | Why Are We Providing Free Intervention? |
|---|---|
| 1. Complete assessment and intervention free of cost<br>2. Post-intervention consultation and advice<br>3. Confidentiality of all personal and medical information | 1. The university is committed to serving the community<br>2. To explore more effective intervention methods<br>3. As part of education and research activities |

| Contact for Free Intervention | |
|---|---|
| Dr. Kazi Md Azman Hossain, PT<br>Physiotherapist, JUST<br>Contact: 01660140833<br>Email: azmanhossain51@gmail.com | Dr. Tofajjal Hossain, PT<br>Physiotherapist, JUST<br>Email: tofajjalhossainphysio@gmail.com |

# 4. Annexure: D – Informed Consent Form

## Children with ASD Consent Form

Assalamu Alaikum / Namaste,

I am Kazi Md Azman Hossain, from the Department of Physiotherapy and Rehabilitation, Jashore University of Science and Technology (JUST), Jashore, Bangladesh.

*I am conducting a research study titled:*

"Effectiveness of a Five-Component Multimodal Intervention on Executive Function in Children with Autism Spectrum Disorder: A Randomized Controlled Trial."

This study has been reviewed and approved by the Institutional Review Board (IRB) of the Department of Physiotherapy and Rehabilitation, JUST (Ethical ID: PTR-JUST/IRB/2025/10/192404) and registered in the Clinical Trials Registry – India (CTRI/2025/11/096943).

**Purpose of the Study**

The aim of this study is to examine the effects of a structured, five-component multimodal exercise program on executive function, social responsiveness, and behavioral characteristics among children with Autism Spectrum Disorder (ASD).

Your child's participation will help develop a safe and effective community-based rehabilitation program for children with ASD in Bangladesh.

**Procedures**

If you agree for your child to participate:

Your child will be randomly assigned to either the intervention group (receiving a 12-week structured exercise program) or the wait-list control group (receiving healthy lifestyle guidance).

Each session will last approximately 60 minutes, 5 times per week for 12 weeks.

Assessments will be conducted before, immediately after, and 12 weeks following the interventions to measure cognitive and behavioural changes.

You may withdraw your child from the study at any time without any consequences.

**Risks and Benefits**

The intervention program is non-invasive and low-risk. Some children may experience mild fatigue during exercises, which will be closely monitored by qualified physiotherapists.

Potential benefits include improved attention, memory, social communication, and overall executive functioning. Participation may also support your child's daily and school activities.

**Confidentiality**

All information and data related to your child will be kept strictly confidential.

Data will be stored securely using unique identification codes, ensuring that personal information is kept confidential and not disclosed.

Photographs or videos, if taken, will have your child's face blurred unless you provide additional written consent.

**Voluntary Participation**

Participation is entirely voluntary.

You are free to refuse participation or withdraw at any stage without affecting your child's ongoing care or rights.

**Contacts for Further Information**

If you have any questions or concerns regarding this study, please feel free to contact:

Kazi Md Azman Hossain

Email: azmanhossain51@gmail.com

Phone: 01660140833

**Consent Declaration**

I have read (or have had read to me) the above information about the study titled -

"Effectiveness of a Five-Component Multimodal Intervention on Executive Function in Children with Autism Spectrum Disorder."

I understand the purpose, procedures, potential risks, and benefits of participation.

I voluntarily agree for my child to participate in this study.

Participant (Child) ID: ____________________________

Child's Name: ____________________________

Parent/Guardian's Name: ____________________________

Parent/Guardian's Signature: ____________________________

Date: ____________________________

Researcher's Name & Signature: ____________________________

## Healthy Children Consent Form

Assalamu Alaikum / Namaste,

I am Kazi Md Azman Hossain, from the Department of Physiotherapy and Rehabilitation, Jashore University of Science and Technology (JUST), Jashore, Bangladesh.

*I am conducting a research study titled:*

"Effectiveness of a Five-Component Multimodal Intervention on Executive Function in Children with Autism Spectrum Disorder: A Randomized Controlled Trial."

This study has been reviewed and approved by the Institutional Review Board (IRB) of the Department of Physiotherapy and Rehabilitation, JUST (Ethical ID: PTR-JUST/IRB/2025/10/192404) and registered in the Clinical Trials Registry – India (CTRI/2025/11/096943).

**Purpose of the Study**

The purpose of this research is to examine the effects of a structured multimodal exercise program on executive function in children with Autism Spectrum Disorder (ASD).

To understand and compare normal developmental levels, we are including a healthy control group consisting of typically developing children who do not have ASD or any other neurological or developmental condition.

Your child's participation will help researchers understand the natural developmental patterns of cognitive and physical functions, supporting the development of better rehabilitation programs for children with ASD.

**Procedures**

Your child will not receive any exercise-based treatment during the study.

Your child will be asked to follow a healthy lifestyle and nutritional guidance provided by the research team throughout the study period.

Your child will take part in three assessment sessions — at baseline, after 12 weeks, and after a 12-week follow-up period — conducted at the Department of Physiotherapy and Rehabilitation, JUST, or an affiliated school facility.

These assessments will include simple cognitive and physical tests, such as attention, memory, and flexibility tasks.

You and your child may withdraw from the study at any time without any consequence or loss of benefits.

**Risks and Benefits**

This study involves minimal to no risk. All activities are safe and non-invasive.

While there may be no direct personal benefit, your child's participation will help researchers establish developmental reference data and contribute to the advancement of therapeutic programs for children with ASD.

Following a healthy lifestyle and receiving nutritional guidance may also encourage positive daily habits that support your child's overall well-being.

**Confidentiality**

All information and data related to your child will be kept strictly confidential.

Data will be stored securely using unique identification codes, ensuring that personal information remains confidential and is not disclosed.

Photographs or videos, if taken, will have your child's face blurred unless you provide additional written consent.

**Voluntary Participation**

Participation is entirely voluntary.

You are free to refuse participation or withdraw at any stage without affecting your child's ongoing care or rights.

**Contacts for Further Information**

If you have any questions or concerns regarding this study, please feel free to contact:

Kazi Md Azman Hossain
Email: azmanhossain51@gmail.com
Phone: 01660140833

**Consent Declaration**

I have read (or have had read to me) the above information about the study titled -
"Effectiveness of a Five-Component Multimodal Intervention on Executive Function in Children with Autism Spectrum Disorder."

I understand the purpose, procedures, potential risks, and benefits of participation.

I voluntarily agree for my child to take part in this study.

# 5. Annexure: E – Questionnaire

**Section I**

Personal details & socio-demographic status

Lifestyle information

Health and Medical Background

**Section II**

***Primary Outcome Measures***

Go/No-Go (GNG) Task

Trail Making Test (TMT)

Corsi Block Tapping Task (CBTT)

Forward Digit Span (FDS) Test

Backward Digit Span (BDS) Test

***Secondary Outcome Measures***

Social Responsiveness Scale, Second Edition (SRS-2)

Autism Treatment Evaluation Checklist (ATEC)

EC Approved Protocol

## 6. Annexure: F – Intervention Tracking Sheet/Checklist
## 7. (Put a tick √)

| Patient ID | Group | Start date | Intervention Timeline<br>Session: 1 to 60 | End date | Any comments |
|---|---|---|---|---|---|
| | | | | | |

## 7. Annexure: G – Adverse Effect Checklist

**(Put a tick √ and write a comment if any adverse effect notices, and put a cross X mark if no adverse effect notice)**

| Patient ID | Group | Pretest | Intervention | Post-test | Follow-up |
|---|---|---|---|---|---|
| | | | | | |

EC Approved Protocol

## 8. Annexure: H – Time Frame: (Gantt Chart)

| Activity | Activity Title | (December 2025 - November 2026) | | | | | | | | | | | |
|---|---|---|---|---|---|---|---|---|---|---|---|---|---|
| | | Dec | Jan | Feb | Mar | Apr | May | Jun | Jul | Aug | Sep | Oct | Nov |
| 1 | Proposal Formulation | | | | | | | | | | | | |
| 2 | Questionnaire Formulation | | | | | | | | | | | | |
| 3 | Ethical Approval | | | | | | | | | | | | |
| 4 | Trial Registration | | | | | | | | | | | | |
| 5 | Publicity (Online & Offline) | | | | | | | | | | | | |
| 6 | Data Collection | | | | | | | | | | | | |
| 7 | Intervention | | | | | | | | | | | | |
| 7 | Data Audit & Analysis | | | | | | | | | | | | |
| 8 | Writing Progression & Completion | | | | | | | | | | | | |
| 9 | Research Submission & Approval | | | | | | | | | | | | |

EC Approved Protocol

| | | | | | | | | | | | | | |
|---|---|---|---|---|---|---|---|---|---|---|---|---|---|
| 10 | Submission for publication | | | | | | | | | | | | |
| 11 | Disseminating through conferences | | | | | | | | | | | | |
| 12 | Disseminating through Seminars | | | | | | | | | | | | |

EC Approved Protocol

## 9. Annexure: I – Budget

| Traits | Costs |
|---|---|
| Questionnaire printing & Measuring tools | 20,000/= |
| Advertisement | 50,000/= |
| Data Collection | 105,000/= |
| Intervention materials | 45,000/= |
| Intervention application | 336,000/= |
| Transport | 50,000/= |
| Meeting/Discussion arrangements | 100,000/= |
| Miscellaneous | 50,000/= |
| **Total** | 756,000/= |
| 5% increment | 6,200/= |
| **Total, including 5% increment** | 793,800/= |

EC Approved Protocol

## 10. Annexure: J – Funding Document

**Dept. of Physiotherapy and Rehabilitation**
**Jashore University of Science and Technology**
Jashore -7408, Bangladesh
Phone: +88 0242142160
Fax: +88 0421 61199, 62238
E-mail: chairmanphysio@gmail.com

**ফিজিওথেরাপি অ্যান্ড রিহ্যাবিলিটেশন বিভাগ**
**যশোর বিজ্ঞান ও প্রযুক্তি বিশ্ববিদ্যালয়**
যশোর-৭৪০৮, বাংলাদেশ।
ফোন: +৮৮ ০২৪২ ১৪২১৬০
ফ্যাক্স: +৮৮ ০৪২১ ৬১১৯৯, ৬২২৩৮

**Reference No: JUST/PTR/2025/17** **Date: September 29, 2025**

### FUNDING APPROVAL LETTER

Kazi Md Azman Hossain
Department of Physiotherapy and Rehabilitation
Jashore University of Science and Technology
Jashore-7408, Bangladesh.

Dear Hossain,
I am pleased to inform you that, after reviewing your study proposal, the academic committee recently approved a grant of **BDT 100,000.00,** which will be released within 1 month. The purpose of this grant is to assist in the data collection and intervention provision for the project titled "Effectiveness of a Five-Component Multimodal Intervention on Executive Function in Children with Autism Spectrum Disorder: A Randomized Controlled Trial."
In all public acknowledgements, we prefer that you indicate this grant as being received from the University Grants Commission of Bangladesh through Jashore University of Science and Technology. The recipients must adhere to the existing taxation policy of the Government of the People's Republic of Bangladesh. We ask that you acknowledge receipt of this grant and indicate your acceptance of the terms discussed above. The grant was approved on September 26, 2025. The grant number is 25-FoHS-17. Please provide this number during the acknowledgement and funding information in your reports and publications. Please note that this grant does not cover journal publication charges, and funds from this grant cannot be used for that purpose. You must also submit a detailed report to the Department of Physiotherapy and Rehabilitation in June 2026, detailing your research project's progression and receipt of the allocated budget. In the meantime, you have our very best wishes for continued success.

Sincerely,

Dr. Ehsanur Rahman
Assistant Professor and Chairman
Department of Physiotherapy and Rehabilitation
Jashore University of Science and Technology (JUST), Jashore-7408, Bangladesh
E-mail: e.rahman@just.edu.bd

ফিজিওথেরাপি অ্যান্ড রিহ্যাবিলিটেশন বিভাগে ফিজিওথেরাপি চিকিৎসা ব্যবস্থা চলমান রয়েছে।
স্বল্প মূল্যে যে কেউ উক্ত বিভাগে ফিজিওথেরাপি চিকিৎসা নিতে পারবেন।
রোগী দেখার সময়: প্রতি শনি-বুধ (সরকারি ছুটি ব্যতিত), সকাল-৯টা থেকে বিকাল-৫টা পর্যন্ত।
যোগাযোগ: এম আর খান মেডিকেল সেন্টার (৩য় তলা), রুম নং-৩১১, মোবাইল: ০১৫৫৩ ৩৩৭ ০৯৪ ।
